# US emergency department visits by women due to assault (2018–2021): a retrospective cross-sectional analysis

**DOI:** 10.1016/j.lana.2025.101343

**Published:** 2026-01-06

**Authors:** Summer Chavez, Irma Ugalde, Michael Ulrich, Omolola Adepoju, Tonghui Xu, Winston Liaw

**Affiliations:** a Tilman J. Fertitta College of Medicine, University of Houston, United States; b Humana Integrated Health Sciences Institute, United States; c University of Chicago Medicine, United States; d Center for Health Law, Ethics, & Human Rights, Boston University School of Public Health, United States; e Boston University School of Law, United States

**Keywords:** Violence against women, Gender-based violence, Healthcare utilization, Disparities, Domestic violence, Intimate partner violence, Firearm injuries, Homicide

## Abstract

**Background:**

Domestic violence has played a key role in linking firearms and homicide amongst female individuals. Combined with the increase of reports of violence against women during the COVID-19 pandemic, a rise in emergency department (ED) visits may be witnessed. Our aim was to estimate the changes in prevalence and risk factors associated with assault and firearm-related emergency department (ED) visits by female patients following the COVID-19 pandemic.

**Methods:**

We performed a retrospective cross-sectional study of female patients presenting to EDs due to assault from the National Emergency Department Sample (NEDS) from 2018 to 2021. Independent variables included age, race, mortality, ED disposition, primary payer, location, mean total ED chargers, quartile ZIP income, and mechanism and intent of injury. The adjusted association between independent variables and ED visits among patients injured by firearms compared to those injured by other assaults was examined.

**Findings:**

The analytic sample represented an estimated 1,575,543 ED weighted records of female assault cases out of a total weighted sample of 537,133,200 observations (0.29%). While year-over-year ED encounters decreased, firearm injuries and the proportion of patients admitted and dying in the hospital increased. Female patients who were injured by firearms had 89 times higher risk of dying in the ED (RR = 88.82; 95% CI 6 = 72.38–97.06) compared to female patients injured by non-firearm injury mechanisms. Racial disparities were prevalent, with Native American women experiencing the greatest risk of being assaulted (RR = 2.81; 95% CI 2.67–2.97). Victims of firearm related assaults had nearly 4.12 times the risk of identifying as Black compared with those assaulted without firearms (95% CI 3.75–4.52). Female patients seeking care for assault had higher risk of being uninsured (95% CI 2.70–2.77).

**Interpretation:**

While year-over-year ED encounters due to assault decreased, lockdowns and restrictions associated with the observed COVID-19 pandemic may not fully reflect changes in abuse rates in this time period. The strong connection between firearm presence and female homicide and continuations of assault and firearm-related ED visits among vulnerable demographic groups highlights the need for effective strategies to reduce violence.

**Funding:**

Unfunded.

**Research in context**

**Evidence before this study**

Domestic violence, which often goes unreported, can lead to the mortality of women given the strong link between firearms and homicide. This threat coincides with the increase in domestic violence experienced under the COVID-19 pandemic lockdown orders. To understand the current literature, we did a comprehensive search of PubMed Central for articles published from January 1, 2012 to March 1, 2025, using the following keywords: “assault” [All Fields] AND “firearms” [All Fields] AND “domestic violence” [All Fields] which yielded 455 articles. These articles individually support the increased risk of firearms and homicide in female patients experiencing abuse, and the rise of domestic violence during and following the pandemic but fail to link them together. We set out to analyze all assault and firearm-linked assault emergency department (ED) visits on an individual demographic level to offer an accurate assessment of domestic violence following the pandemic.

**Added value of this study**

This study uses data from the National Emergency Department Sample (NEDS) to provide a detailed breakdown of the changes in the frequency of ED visits for both assault and firearms-linked assault cases from 2018 to 2021. Because NEDS is a record-level rather than linked by individuals, we are unable to determine whether records represent the same individual. In tracking and comparing the changes between trends in both other assault and firearm-related assault ED visits, this study helps to determine the association between firearm presence and female homicide following the COVID-19 pandemic and associations of individual patient-level demographics with these all-assault visits.

**Implications of all the available evidence**

The findings of this study provide essential insights for lawmakers, legislatures, and those working in domestic violence services. In analyzing ED visit rates for all assault and firearms-linked assault cases, this work demonstrates an association between firearm presence and female homicide. This makes the findings particularly valuable to inform policy makers, community members and organizations in creating safer environments in the United States, especially among female populations that are at a heightened risk of experiencing domestic violence.

## Introduction

In the United States, 1 in 4 women aged 18 and older are victims of severe physical violence by an intimate partner (between romantic partners but not necessarily living together) while up to half of women experience domestic violence (occurring between individuals living in the same household).^1^ Additionally, one in five women has experienced an attempted or completed rape in their lifetime.^2^ During the COVID-19 pandemic, pooled estimates of women experiencing any violence were 21% (95% CI 18–23%).^3^ In emergency medicine settings, the lifetime prevalence of any type of intimate partner violence (IPV) is as high as 40%.^4^ All estimates are likely underrepresented because women are still hesitant to report instances of assault and violence due to stigma, shame, fear of retaliation, fear of negative reactions, feelings of guilt and embarrassment, concerns about evidence, and protection of others (e.g. family members).^5^ As a result, accurately determining the true prevalence of violence against women, contributing etiologies and understanding the needs of this population remains a significant challenge, making it increasingly difficult to develop effective strategies and allocate adequate resources for prevention, recovery, and support.

A better understanding of the role firearms play in domestic violence is especially important given the associations between firearms and homicide. Evidence suggests a woman is eleven times more likely to be killed by their abuser if a firearm is present.^6^ The CDC reports that in 2020, intimate partner violence precipitated approximately 41.5% of homicides for women, but only 7.2% for men.^7^ In instances where IPV is a contributing factor and the relationship is known, 93.5% of female homicides are committed by a suspect who is a current or former intimate partner.^7^ There are broader implications for the public as well. Between 2014 and 2019, 59.1% of mass shootings were tied to domestic violence.^8^ Up to 14% of female patients admitted to the emergency department are treated for conditions caused by either intimate partner violence or domestic violence. In addition to this, between 5% and 38% of all women seen in the emergency department report experiencing intimate partner violence in the last year.^9^ After pandemic-related lockdown orders were implemented in the United States, domestic violence incidents increased by roughly 8%.^10^ In some areas, domestic violence calls to police increased by as much as 27% in March 2020 compared to March 2019.^11^ Research examining data from emergency departments (EDs) and trauma centers has reinforced these findings. When examining a level 1 trauma center, researchers found that the odds of penetrating violence (i.e. firearms, knives) against women were 5 times greater during the pandemic.^12^

The aim was to estimate the changes in prevalence and risk factors associated with assault and firearm-related emergency department (ED) visits by female patients following the COVID-19 pandemic. This study contributes to the understanding of patterns of violence towards female patients before and during the COVID-19 pandemic in the US, with particular attention to disparities and firearm involvement.

## Methods

This was a retrospective cross-sectional study of female patients presenting to US EDs due to assault from 2018- to 2021. Our study sample included ED visit data from the National Emergency Department Sample (NEDS) during the study period. NEDS is the largest all-payer database of ED encounters, derived from hospital billing data as part of the Healthcare Cost and Utilization Project (HCUP) at the Agency for Healthcare Research and Quality.^13^ This data source includes information about patient and hospital demographics and clinical encounter-specific data. NEDS stratifies by geography, trauma designation, urban/rural location, teaching status and hospital ownership/control to create a sample representative of all US hospitals. NEDS represents approximately 30 million ED visits from 993 hospitals, totaling an estimated 127 million visits annual and covering 84% of the US population. From the discharge summaries, medical coders will assign diagnosis and procedure codes.^14^ Due to the complex nature of survey data and the need for precise estimates, we applied weighting to ensure that the results are representative and properly reflect the population structure. Observations that met the exclusion criteria were used to calculate standard errors. Only observations that meet the inclusion criteria are used to calculate estimates. HCUP performs internal quality control procedures and data checks to ensure the accuracy of data from participating healthcare organizations.

Our exclusion criteria were aged <18 years, male sex, and injury of intent other than assault (i.e. unintentional or self-harm).We collected information related to patient characteristics including age (years), race (White, Black, Hispanic, Asian or Pacific Islander, Native American, Other), mortality (did not die, death in hospital or ED), ED disposition (home/discharge, admitted, transferred, died in ED), primary payer (Medicare, Medicaid, private, uninsured), location (metropolitan/non-metropolitan), mean total ED charges ($ USD), median household income for patient ZIP code by quartile, mechanism of injury and intent of injury (assault, unintentional, self-harm). Determination of metropolitan or non-metropolitan was based on the National Center for Health Statistics (NCHS) Urban-Rural Code and the 2023 Rural-Urban Continuum Codes (RUUC), where counties in metropolitan areas of 50,000–249,999 population were recoded as metropolitan; micropolitan counties and those counties that were not micropolitan or metropolitan were recoded as non-metropolitan.^15^ The mechanism of injury is based on External Cause of Injury Codes and includes cutting/piercing, falling, fire, firearm, machinery, motor vehicle traffic, natural/environmental, poisoning, suffocation, and being struck by or against, drowning, or machinery. Due to small sample sizes, drowning and machinery injuries were combined. The final analytic ED visit sample was 375,324. The analysis was conducted on complete cases for each model. The missing-data rate was 4%. Given the low prevalence of missingness, we relied on the default handling of missing data by statistical software (complete-case analysis), which is unlikely to substantially bias the results.

We describe the epidemiologic characteristics of female assault patients using descriptive statistics for annual cross-sectional data over a four-year period. Next, we combined all study year data to calculate national estimates of total ED visits and ED visits related to assault across this period. For categorical variables, we used the Chi^2^ test; for continuous variables, we fit unadjusted simple regression models to compare mean age over time. We initially employed a survey-weighted binary logistic regression model. However, several results were affected by sparse-data bias and quasi-complete separation, leading to inflated odds ratio estimates. Consequently, we adopted a more robust approach by estimating relative risks (RRs) using a survey-weighted generalized linear model (GLM) with a Poisson distribution and a log link. This model assessed the risk of assault among female patients relative to all female patients visiting the ED. The results from the initial survey-weighted logistic regression are provided in the Supplement for comparison. The first regression model adjusted for age in years, calendar year, death, payer, metropolitan status, race and median household income for patient ZIP code quartile. The second regression model used the same covariates but compared female assault patients injured by firearms compared to all other mechanisms of assault. We repeated these two regression models by age category (18–29 years, 30–39 years, 40–49 years, 50+ years). Stata 18.0 (College Station, Texas) was used. This study was reviewed by University of Houston (STUDY00004762) and deemed Not Human Subjects research. All primary analyses accounted for the complex sampling design, weighting structure, clusters, and currency inflation (all reported in 2021 U.S. dollars) of the NEDS data when generating respective national estimates reported in this study.

### Role of the funding source

There were no study sponsors involved in the study design, analysis or interpretation of data, the writing of the manuscript, or the decision to submit the manuscript for publication. The authors were not precluded from accessing data in the study. All authors accept responsibility to submit for publication. There was no publication fee to write this article by a pharmaceutical company or any other agency.

## Results

**Fig. 1:**
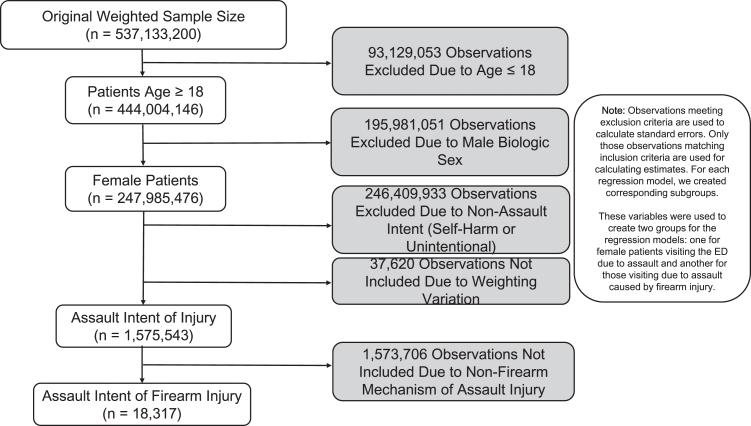
Flowchart of the study sample.

The national estimate of US ED encounters from 2018- to 2021 was 537,133,200 observations. After applying weighting exclusions, the final estimated number of national ED visits related to female assault was 1,575,543 (0.29%) observations across the four-year period (Fig. 1). Table 1 shows the unweighted descriptive statistics of our sample of visits due to female assault (n = 375,324). The estimated firearm assault population was 18,317. We report annual estimated ED visits counts for each study year, patient characteristics and hospital information. Over the study period, annual visits due to assault for female patients decreased. 69% (n = 1,082,044) of the sample were women 18–39 years of age. 46% (n = 517,761) of the study population was White, while Black female patients accounted for 32% (n = 363,700) of all patients. Each year the frequency of patients dying in the hospital and proportion of all female patients admitted to the hospital increased. 43% (n = 160,848) of the sample size was insured by Medicaid, while another 29% (n = 108,907) was uninsured or self-pay. 43% (n = 156,925) of the women in the study sample had median incomes for their ZIP codes in the 0–25th percentile. The most common mechanisms of injury were being struck by or against, cutting/piercing, falling, firearm injuries or suffocation. Firearm injuries were the only mechanism type that consistently increased each year. Notably, suffocation injuries increased in 2020; nearly every other injury type except firearm injuries decreased. Multiple mechanisms of injury increased during the pandemic compared to prior years (Table 2).

**Table 1:** Unweighted clinical characteristics of female patients visiting the ED due to assault from 2018 to 2021 in the United States.

	2018	2019	2020	2021	2018–2021
**Visit counts**	105,533 (28%)	100,140 (27%)	87,592 (23%)	82,059 (21%)	375,324 (100%)
**Mean age (Years)**	35.4	35.2	35.6	35.8	35.4
**Age category**					
18–29 years	45,678 (43%)	42,828 (43%)	36,046 (41%)	33,091 (40%)	157,643 (42%)
30–39 years	27,406 (26%)	26,534 (27%)	23,590 (27%)	22,281 (27%)	99,811 (27%)
40–49 years	15,900 (15%)	15,083 (15%)	13,645 (16%)	13,085 (16%)	57,713 (15%)
50+ years	16,547 (15%)	15,676 (16%)	14,309 (16%)	13,597 (17%)	60,129 (16%)
**Race**					
White (1)	N/A	44,975 (46%)	39,173 (47%)	35,316 (45%)	119,464 (46%)
Black (2)	N/A	32,272 (33%)	27,262 (32%)	24,762 (32%)	84,296 (32%)
Hispanic (3)	N/A	14,565 (14%)	12,180 (14%)	13,356 (16%)	40,101 (15%)
Asian/Pacific Islander (4)	N/A	1464 (1.5%)	1638 (1.9%)	1656 (2%)	4758 (1.8%)
Native American (5)	N/A	1308 (1.4%)	1549 (2.0%)	1454 (1.9%)	4311 (1.7%)
Other (6)	N/A	3603 (3.6%)	3208 (3.9%)	2803 (3.7%)	9614 (3.7%)
**Death**					
Did not die	105,183 (99.86%)	99,863 (99.84%)	87,321 (99.70%)	81,787 (99.67%)	374,154 (99.81%)
Died in ED	60 (0.06%)	61 (0.06%)	72 (0.08%)	69 (0.08%)	262 (0.07%)
Died in hospital	86 (0.08%)	99 (0.1%)	120 (0.22%)	140 (0.25%)	445 (0.12%)
**Disposition from ED**					
Home/Discharge (1,98,99)	99,645 (94.42%)	94,100 (93.97%)	81,175 (92.68%)	75,982 (92.59%)	350,902 (93.49%)
Admitted (2)	4789 (4.54%)	4776 (4.77%)	5166 (5.90%)	5077 (6.19%)	19,808 (5.28%)
Transferred (3)	1039 (0.98%)	1203 (1.20%)	1179 (1.34%)	931 (1.14%)	4352 (1.16%)
Died in ED (9)	60 (0.06%)	61 (0.06%)	72 (0.08%)	69 (0.08%)	262 (0.07%)
**Primary payer**					
Medicare (1)	9604 (9.2%)	9001 (9.0%)	8293 (9.6%)	7404 (9.2%)	34,302 (9.2%)
Medicaid (2)	45,123 (43%)	40,690 (41%)	37,621 (43%)	37,414 (46%)	160,848 (43%)
Private (3)	19,192 (18%)	18,817 (19%)	16,620 (19%)	15,769 (19%)	70,398 (19%)
Self-Pay, no charge, other (4/5/6)	31,403 (29%)	31,381 (31%)	24,832 (28%)	21,291 (26%)	108,907 (29%)
**Patient location**					
Metropolitan (1, 2, 3, 4)	90,114 (85%)	85,309 (86%)	72,953 (85%)	68,500 (85%)	316,876 (85%)
Non-Metropolitan (5,6)	13,705 (15%)	13,445 (14%)	12,271 (15%)	11,553 (15%)	50,974 (15%)
**Mean total ED charges($)**	5169.6	5824.8	6572.7	6556.1	6009.5
**Median household income for patient's ZIP code**					
0–25th percentile (1)	44,639 (44%)	42,027 (43%)	36,437 (43%)	33,822 (42%)	156,925 (43%)
26–50th percentile (2) median	27,113 (27%)	24,474 (25%)	22,135 (26%)	19,521 (25%)	93,243 (26%)
51–75th percentile (3)	17,794 (17%)	18,704 (19%)	14,919 (18%)	15,107 (19%)	66,524 (18%)
76–100th percentile (4)	12,749 (12%)	12,236 (13%)	10,573 (13%)	10,527 (14%)	46,085 (13%)
**Mechanism of Injury**					
Cutting or piercing	3694 (3.5%)	3514 (3.5%)	3465 (3.9%)	2977 (3.6%)	13,650 (3.6%)
Fall	1467 (1.4%)	1526 (1.5%)	1409 (1.7%)	1329 (1.6%)	5812 (1.6%)
Fire, flame, hot object, or hot substance	267 (0.3%)	228 (0.2%)	206 (0.2%)	201 (0.3%)	902 (0.2%)
Firearm	956 (0.9%)	997 (1.0%)	1212 (1.3%)	1244 (1.5%)	4409 (1.2%)
Motor vehicle traffic, occupant of car, motorcyclist, pedal cyclist, pedestrian or other	527 (0.5%)	475 (0.5%)	486 (0.5%)	488 (0.6%)	1976 (0.5%)
Natural or environmental, including bites & stings	216 (0.2%)	224 (0.2%)	223 (0.3%)	193 (0.2%)	856 (0.2%)
Overexertion	118 (0.1%)	147 (0.2%)	136 (0.2%)	115 (0.1%)	516 (0.1%)
Poisoning including drugs and non-drugs	118 (0.9%)	147 (0.9%)	136 (1.0%)	115 (1.1%)	516 (1.0%)
Struck by or against	950 (68%)	860 (69%)	870 (65%)	924 (65%)	3604 (67%)
Suffocation	(0.4%)	(0.7%)	(1.6%)	(1.6%)	(1.0%)
**Multiple mechanisms of injury**					
1	75,802 (97.32%)	73,058 (96.98%)	60,865 (95.80%)	56,843 (95.96%)	266,568 (96.58%)
2	2054 (2.64%)	2245 (2.98%)	2627 (4.13%)	2355 (3.98%)	9281 (3.36%)
3+	29 (0.04%)	30 (0.08%)	43 (0.07%)	40 (0.06%)	142 (0.06%)

Note. Since the sample contains missing values, the sum of frequencies for some variables (including age, race, death, primary payer, patient location, mean total ED charges, and median household income for the patient's ZIP code) may not equal the final analytic sample.

**Table 2:** Weighted clinical characteristics of female patients visiting the ED due to assault from 2018 to 2021 in the United States.

	2018	2019	2020	2021	P-value	2018–2021
**Visit counts**	416,759 (26%)	431,367 (27%)	380,706 (24%)	346,711 (22%)	–	1,575,543 (100%)
**Mean age**^a^**(Years)**	35.1 ± 0.04	35.2 ± 0.05	35.6 ± 0.05	35.8 ± 0.05	<0.0001	35.4 ± 0.02
**Age category**^c^					<0.0001	
18–29 years	181,085 (43%)	184,681 (43%)	157,194 (41%)	139,924 (40%)		662,885 (42%)
30–39 years	108,310 (26%)	114,567 (27%)	102,315 (27%)	93,968 (27%)		419,159 (27%)
40–49 years	62,788 (15%)	64,927 (15%)	59,195 (16%)	55,272 (16%)		242,182 (15%)
50+ years	64,569 (15%)	67,091 (16%)	61,995 (16%)	57,527 (17%)		251,181 (16%)
**Race**^b^					<0.0001	
White (1)	N/A	194,099 (46%)	172,764 (47%)	150,898 (45%)		517,761 (46%)
Black (2)	N/A	140,982 (33%)	116,849 (32%)	105,868 (32%)		363,700 (32%)
Hispanic (3)	N/A	59,536 (14%)	51,187 (14%)	52,996 (16%)		163,720 (15%)
Asian/Pacific Islander (4)	N/A	6458 (1.5%)	6931 (1.9%)	6785 (2%)		20,175 (1.8%)
Native American (5)	N/A	5846 (1.4%)	7228 (2.0%)	6506 (1.9%)		19,579 (1.7%)
Other (6)	N/A	15,275 (3.6%)	14,292 (3.9%)	12,457 (3.7%)		42,024 (3.7%)
**Death**^c^					<0.0001	
Did not die	415,301 (100%)	430,173 (100%)	379,565 (100%)	345,563 (100%)		1,570,602 (100%)
Died in ED	229 (0.0%)	278 (0.0%)	311 (0.0%)	283 (0.0%)		1101 (0.0%)
Died in hospital	334 (0.0%)	436 (0.1%)	506 (0.1%)	601 (0.2%)		1877 (0.1%)
**Disposition from ED**^c^					<0.0001	
Home/Discharge (1,98,99)	393,715 (94%)	405,458 (94%)	353,375 (93%)	320,993 (93%)		1,473,541 (94%)
Admitted (2)	18,423 (4.4%)	20,297 (4.7%)	21,897 (5.8%)	21,443 (6.2%)		82,061 (5.2%)
Transferred (3)	4392 (1.1%)	5334 (1.2%)	5123 (1.3%)	3992 (1.2%)		18,841 (1.2%)
Died in ED (9)	229 (0.0%)	278 (0.0%)	311 (0.0%)	283 (0.0%)		1101 (0.1%)
**Primary payer**^c^					<0.0001	
Medicare (1)	38,138 (9.2%)	38,671 (9.0%)	36,293 (9.6%)	31,661 (9.2%)		144,764 (9.2%)
Medicaid (2)	178,499 (43%)	174,575 (41%)	162,426 (43%)	158,527 (46%)		674,027 (43%)
Private (3)	76,812 (18%)	81,490 (19%)	73,210 (19%)	66,117 (19%)		297,629 (19%)
Self-Pay, no charge, other (4/5/6)	122,449 (29%)	135,461 (31%)	107,839 (28%)	89,559 (26%)		455,308 (29%)
**Patient location**^c^					<0.0001	
Metropolitan (1, 2, 3, 4)	347,474 (85%)	364,329 (86%)	313,648 (85%)	287,513 (85%)		1,312,964 (85%)
Non-Metropolitan (5,6)	63,378 (15%)	61,705 (14%)	56,819 (15%)	50,576 (15%)		232,478 (15%)
**Mean total ED Charges**^b^**($)**	5741 ± 29	6327 ± 27	6726 ± 30	6322 ± 31		6286 ± 14
**Median household income for patient's ZIP code**^c^					<0.0001	
0–25th percentile (1)	177,099 (44%)	181,391 (43%)	157,308 (43%)	140,782 (42%)		656,580 (43%)
26–50th percentile (2) Median	109,543 (27%)	104,543 (25%)	96,185 (26%)	82,233 (25%)		392,504 (26%)
51–75th percentile (3)	69,272 (17%)	81,043 (19%)	65,731 (18%)	64,742 (19%)		280,787 (18%)
76–100th percentile (4)	49,011 (12%)	53,422 (13%)	46,680 (13%)	45,857 (14%)		194,969 (13%)
**Mechanism of injury**^c^						
Cutting or piercing	14,559 (3.5%)	15,110 (3.5%)	14,845 (3.9%)	12,489 (3.6%)	<0.0001	57,003 (3.6%)
Fall	5772 (1.4%)	6684 (1.5%)	6455 (1.7%)	5679 (1.6%)	<0.0001	24,590 (1.6%)
Fire, flame, hot object, or hot substance	1037 (0.3%)	941 (0.2%)	888 (0.2%)	862 (0.3%)	0.48	3729 (0.2%)
Firearm	3638 (0.9%)	4448 (1.0%)	4929 (1.3%)	5301 (1.5%)	<0.0001	18,317 (1.2%)
Motor vehicle traffic, occupant of car, motorcyclist, pedal cyclist, pedestrian or other	2038 (0.5%)	2055 (0.5%)	2019 (0.5%)	1975 (0.6%)	0.030	8086 (0.5%)
Natural or environmental, including bites & stings	848 (0.2%)	962 (0.2%)	973 (0.3%)	847 (0.2%)	0.11	3631 (0.2%)
Overexertion	485 (0.1%)	642 (0.2%)	602 (0.2%)	487 (0.1%)	0.11	2217 (0.1%)
Poisoning including drugs and non-drugs	3753 (0.9%)	3731 (0.9%)	3683 (1.0%)	3899 (1.1%)	<0.0001	15,065 (1.0%)
Struck by or against	284,353 (68%)	298,420 (69%)	247,821 (65%)	223,755 (65%)	<0.0001	1,054,349 (67%)
Suffocation	1741 (0.4%)	2912 (0.7%)	6025 (1.6%)	5615 (1.6%)	<0.0001	16,292 (1.0%)
**Multiple mechanisms of injury**					<0.0001	
1	301,614 (97%)	316,090 (97%)	264,890 (96%)	240,298 (96%)		1,122,892 (97%)
2	8142 (2.6%)	9729 (3.0%)	11,405 (4.1%)	10,080 (4.0%)		39,356 (3.4%)
3+	117 (0.0%)	130 (0.0%)	190 (0.1%)	157 (0.1%)		594 (0.1%)

a Unadjusted linear regression model, modeling age in years over calendar year. Coefficient = 0.27, 95% confidence interval [0.23, 0.32].

b Unadjusted linear regression model, modeling ED charges in $ over calendar year. Coefficient = 413.33, 95% confidence interval [388.46, 438.21].

c Chi-squared.

Compared with the inflated odds ratio estimates of death in the firearm-related model from the survey-weighted logistic regression (Supplementary Table S1 and Table 2), the survey-weighted Poisson regression (Tables 3 and 4) also produced high estimates, but in a more robust manner. Both models, however, yielded results in the same direction, and importantly, the substantive conclusion did not change: the association remained statistically significant across all models.

**Table 3:** Adjusted survey-weighted GLM regression with Poisson distribution and log link assessing the relative risk of female adult patients visiting the emergency department due to (a) all-cause assault compared to the general adult female ED population and (b) firearm-related assault compared to all other adult female assault patients.

Variable	Risk of female adult patients visiting the emergency department due to assault compared to general adult female ED population visiting the emergency department		Risk of female adult patients visiting the emergency department due to firearm injury compared to adult female ED population visiting the emergency department for assault	
	RR	95% Confidence interval	RR	95% Confidence interval
*Age*	0.97	0.97, 0.97^a^	0.98	0.97, 0.98
*Year*	0.97	0.97, 0.97^b^	1.19	1.14, 1.24
*Quartile of median income ZIP code*				
1st	1.11	1.09, 1.12	1.78	1.56, 2.04
2nd	0.96	0.94, 0.97	1.33	1.15, 1.54
3rd	0.96	0.95, 0.98	1.24	1.07, 1.45
4th	REF	REF	REF	REF
*Death*				
In ED	1.04	0.89, 1.21	83.82	72.38, 97.06
In Hospital	1.33	1.19, 1.49	3.34	2.79, 4.00
Did not die	REF	REF	REF	REF
*Payer*				
Medicare	1.23	1.21, 1.25	0.41	0.33, 0.49
Medicaid	2.04	2.02, 2.07	0.81	0.74, 0.89
Uninsured/Self-Pay/Other	2.74	2.70, 2.77	0.88	0.80, 0.97
Private	REF	REF	REF	REF
*Location classification*				
Metropolitan	REF	REF	REF	REF
Non-Metropolitan	0.82	0.81, 0.83	1.00	0.89, 1.12
*ED disposition*				
Did not die	REF	REF	REF	REF
Admit	0.52	0.52, 0.53	16.50	15.29, 17.80
Transfer	1.16	1.12, 1.20	8.11	6.97, 9.44
Die	–	–	–	–
*Race*				
White	REF	REF	REF	REF
Black	1.29	1.28, 1.30	4.12	3.75, 4.52
Hispanic	0.81	0.80, 0.82	1.87	1.64, 2.12
Asian/Pacific Islander	0.97	0.94, 1.00	0.98	0.66, 1.45
Native American	2.68	2.60, 2.77	0.95	0.64, 1.41
Other	1.08	1.06, 1.11	1.90	1.55, 2.33

RR, relative risk; REF, reference group.

a The original 95% confidence interval is 0.9692–0.9698.

b The original 95% confidence interval is 0.9652–0.9746.

**Table 4:** Adjusted survey-weighted GLM regression with poisson distribution and log link assessing the relative risk of female adult patients visiting the emergency department due to a) all-cause assault compared to general adult female ED population and b) assault by firearm compared to all other adult female assault patients by age.

Variable	Adjusted RR for ED visit due to assault (95% CI)	Adjusted RR for ED visit due to firearm injury (95% CI)	Adjusted RR for ED visit due to assault (95% CI)	Adjusted RR for ED visit due to firearm injury (95% CI)	Adjusted RR for ED visit due to assault (95% CI)	Adjusted RR for ED visit due to firearm injury (95% CI)	Adjusted RR for ED visit due to assault (95% CI)	Adjusted RR for ED visit due to firearm injury (95% CI)
	18–29 years		30–39 years		40–49 years		50+ years	
*Age*	0.99 (0.98, 0.99)	0.96 (0.95, 0.98)	0.98 (0.98, 0.99)	0.99 (0.96, 1.01)	0.97 (0.96, 0.97)	0.98 (0.94, 1.01)	0.95 (0.94, 0.95)	0.96 (0.94, 0.97)
*Year*	0.95 (0.95, 0.96)	1.20 (1.13, 1.27)	0.97 (0.96, 0.98)	1.18 (1.09, 1.29)	0.99 (0.98, 1.00)	1.21 (1.07, 1.36)	0.99 (0.98, 1.00)	1.11 (0.97, 1.27)
*Quartile of median income ZIP code*								
1st	1.01 (0.99, 1.03)	1.73 (1.44, 2.08)	1.17 (1.13, 1.20)	1.54 (1.19, 2.01)	1.15 (1.11, 1.19)	2.42 (1.61, 3.64)	1.17 (1.13 1.211)	1.87 (1.22, 2.86)
2nd	0.91 (0.89, 0.93)	1.28 (1.05, 1.57)	0.99 (0.96, 1.02)	1.18 (0.89, 1.58)	0.98 (0.96 1.02)	1.63 (1.05, 2.53)	0.96 (0.92 0.99)	1.56 (0.99, 2.46)
3rd	0.94 (0.92, 0.96)	1.34 (1.09, 1.65)	0.98 (0.95, 1.01)	1.11 (0.82, 1.50)	0.95 (0.92 0.99)	0.91 (0.55, 1.50)	0.96 (0.93, 0.99)	1.36 (0.84, 2.19)
4th	REF	REF	REF	REF	REF	REF	REF	REF
*Death*								
In ED	3.14 (2.45, 4.02)	77.63 (62.60, 96.27)	1.90 (1.44, 2.50)	78.40 (59.30, 103.65)	0.71 (0.45, 1.13)	92.63 (66.98, 128.10)	0.46 (0.33, 0.63)	110.20 (70.04, 173.38)
In Hospital	4.96 (3.75, 6.57)	3.29 (2.57, 4.22)	1.56 (1.10, 2.22)	4.04 (3.02, 5.40)	0.88 (0.59, 1.31)	4.51 (2.51, 8.11)	1.18 (1.03, 1.36)	2.75 (1.60, 4.70)
Did not die	REF	REF	REF	REF	REF	REF	REF	REF
*Payer*								
Medicare	1.45 (1.38, 1.52)	0.66 (0.45, 0.99)	1.96 (1.88, 2.04)	0.29 (0.18, 0.47)	1.95 (1.88, 2.04)	0.39 (0.25, 0.60)	1.61 (1.56, 1.67)	0.39 (0.27, 0.54)
Medicaid	1.58 (1.55, 1.61)	0.96 (0.85, 1.09)	2.35 (2.30, 2.41)	0.81 (0.67, 0.97)	2.63 (2.55, 2.70)	0.62 (0.48, 0.80)	2.68 (2.60, 2.77)	0.42 (0.30, 0.58)
Uninsured/Self-Pay/Other	2.16 (2.12, 2.20)	1.00 (0.87, 1.15)	3.09 (3.01, 3.17)	0.89 (0.73, 1.09)	3.18 (3.09, 3.28)	0.68 (0.52, 0.90)	3.54 (3.32, 3.66)	0.62 (0.45, 0.86)
Private	REF	REF	REF	REF	REF	REF	REF	REF
*Location classification*								
Metropolitan	0.84 (0.83, 0.86)	0.93 (0.79, 1.09)	0.85 (0.83, 0.87)	0.96 (0.77, 1.20)	0.82 (0.79, 0.84)	0.94 (0.69, 1.29)	0.73 (0.70, 0.75)	1.55 (1.13, 2.13)
Non-Metropolitan	REF	REF	REF	REF	REF	REF	REF	REF
*ED disposition*								
Did not die	REF	REF	REF	REF	REF	REF	REF	REF
Admit	0.48 (0.46, 0.50)	19.94 (18.05, 22.02)	0.48 (0.46, 0.50)	15.96 (13.78, 18.48)	0.48 (0.46, 0.51)	12.38 (10.01, 15.30)	0.64 (0.63, 0.66)	9.71 (7.40, 12.74)
Transfer	1.62 (1.53, 1.71)	8.18 (6.66, 10.04)	1.32 (1.23, 1.42)	9.27 (6.98, 12.30)	1.09 (0.99, 1.19)	6.82 (4.24, 10.95)	09.74 (0.68, 0.80)	5.93 (3.34, 10.54)
Die	–	–	–	–	–	–	–	–
*Race*								
White	REF	REF	REF	REF	REF	REF	REF	REF
Black	1.53 (1.51, 1.55)	4.16 (3.61, 4.78)	1.20 (1.18, 1.23)	4.53 (3.80, 5.41)	1.04 (1.01, 1.06)	3.80 (2.98, 4.85)	1.15 (1.13, 1.19)	3.51 (2.72, 4.53)
Hispanic	0.96 (0.94, 0.97)	1.78 (1.48, 2.14)	0.77 (0.76, 0.79)	1.75 (1.34, 2.28)	0.66 (0.64, 0.68)	2.45 (1.78, 3.36)	0.68 (0.66, 0.71)	2.03 (1.38, 2.99)
Asian/Pacific Islander	0.92 (0.87, 0.97)	1.02 (0.57, 1.85)	0.90 (0.85, 0.95)	0.88 (0.40, 1.92)	0.90 (0.83, 0.97)	0.68 (0.21, 2.16)	1.19 (1.13, 1.27)	1.29 (0.55, 3.05)
Native American	2.81 (2.67, 2.97)	1.24 (0.72, 2.14)	2.66 (2.51, 2.81)	1.10 (0.56, 2.16)	2.47 (2.29, 2.66)	0.57 (0.18, 1.80)	2.29 (2.10, 2.50)	–
Other	1.21 (1.17, 1.25)	2.01 (1.51, 2.68)	0.98 (0.94, 1.03)	2.49 (1.74, 3.57)	0.97 (0.91, 1.02)	1.48 (0.73, 2.98)	1.17 (1.10 1.23)	0.68 (0.27, 1.69)

Table 3 shows the RR of female adult patients visiting the emergency department due to assault compared to general adult female ed population visiting the emergency department. Each increase in age group was associated with a 3% reduction in the risk of the assault (RR = 0.97, CI 0.97–0.97). Female assault patients had 1.33 times greater risk of dying in the hospital compared to all other female patients (95% CI 1.19–1.49). When examining primary payer, female assault patients had more than twice the risk of being insured by Medicaid (RR = 2.04; 95% CI 2.02–2.07) and 2.74 times the risk of being uninsured, self-pay, or other (RR = 2.74; 95% CI 2.70–2.77) compared to the reference group. Differences by race were also evident. Native American patients had the highest risk of assault (RR = 2.68; 95% CI 2.60–2.77), followed by Black patients (RR = 1.29; 95% CI 1.28–1.30), whereas Hispanic patients had a lower odd of experiencing assault (RR = 0.81; 95% CI 0.80–0.82).

In the firearm injury model for women, each increase in age group was associated with a 2% reduction in the risk of assault-related ED visits (RR = 0.98, 95% CI 0.97–0.98). Female patients with firearm injuries had far greater risk of dying in the ED (RR = 77.63; 95% CI 62.60–96.27) or hospital (RR = 3.29; 95% CI 2.57–4.22). They also had substantially higher risk of being admitted to the hospital (RR = 19.94; 95% CI 18.04–22.02). Compared to White female patients with firearm injuries, Black (RR = 4.15; 95% CI 3.61–4.78), Hispanic (RR = 1.77; 95% CI 1.48–2.13) and Other (RR = 2.01; 95% CI 1.51–2.68) patients had greater risk of firearm injury (Table 3).

We re-ran 8 survey-weighted Poisson regression models by age category to examine the most impactful factors in Table 4. Female patients 18–29 years old, had the greatest risk of death, whether in the ED (RR = 3.14; 95% CI 2.45–4.20) or in-hospital (RR = 4.96; 95% CI 3.75–6.57). There appeared to be a bimodal distribution of increased mortality for firearm injuries for 18–29 years (death in ED: RR = 77.65; 95% CI 62.60–96.27, death in hospital: RR = 3.29; 95% CI 2.57–4.22) and 40–49 years (death in ED: RR = 92.63; 95% CI 66.98–128.10, death in hospital: RR = 4.51; 95% CI 2.51–8.11).

As age increased, the risk of being uninsured (18–29 years: RR = 2.16; 95% CI 2.12–2.20, 30–39 years: RR = 3.09; 95% CI 3.01–3.17, 40–49 years: RR = 3.18; 95% CI 3.09–3.28, 50+ years: RR = 3.54; 95% CI 3.42–3.66) or insured by Medicaid (18–29 years: RR = 1.58; 95% CI 1.55–1.61, 30–39 years: RR = 2.35; 95% CI 2.30–2.41, 40–49 years: RR = 2.63; 95% CI 1.87–2.04, 50+ years: RR = 2.68; 95% CI 2.69–2.77, respectively) increased.

Female patients 18–29 years had the greatest risk of admission (RR = 19.94; 95% CI 18.05–22.02). When examining encounters for all assault, Native American female patients 18–29 years had the greatest risk of injury among all races or age categories (RR = 2.81; 95% CI 2.67–2.97) and in general, Native American female patients had the greatest risk of assault compared to any other racial or ethnic group. Black female patients consistently had the greatest risk of firearm injury for every age category compared to every other racial group (Table 4).

## Discussion

In this study analyzing ED visits by female patients experiencing assault from 2018- to 2021, we found several notable trends. First, while yearly ED encounters decreased, firearm injuries increased. Similarly, the proportion of patients admitted to the hospital and dying in the hospital increased each year, reinforcing the lethality of firearms and the increased risk they pose for domestic violence victims. Female patients who were injured by firearms specifically had 379.00 (95% CI 266.15–539.7) times higher risk of dying in the ED compared to all-cause female assault patients. Racial disparities were prevalent in our findings, with Native American women experiencing the greatest risk of being assaulted and victims of firearm-related assaults nearly five times more likely to identify as Black than those assaulted without firearms. The female patients seeking care for assault had a greater risk of being younger and either enrolled in Medicaid or uninsured.

During the COVID-19 pandemic, reports of violence against women increased significantly. After pandemic-related lockdown orders were implemented in the United States, domestic violence incidents increased by roughly 8%.^16^ In some areas, domestic violence calls to police increased by as much as 27% in March 2020 compared to March 2019.^17^ Research examining data from emergency departments and trauma centers reinforced these findings, with one study at a single Level 1 trauma center reporting that the risk of penetrating violence against women were five times greater during the pandemic.^12^ In a similar study conducted in an emergency department, researchers found that the number of cases of violence against women in the emergency department increased 13% during the pandemic period, and the ratio of these cases to all emergency department admissions tripled compared to the pre-pandemic period.^18^ This increase in proportion may be attributed to several factors, including increased exposure to and isolation with abusers, rising unemployment, heightened stress associated with childcare, increased consumption of alcohol and other substances, financial insecurity, and fewer opportunities for others to detect and report signs of abuse before presenting to the ED.^11^^,^^19^ Although our analysis found total ED visits due to assault decreased over time, we did find concerning increases in firearm injuries. Because the data sample is aggregated, this may overlook regional variation.^15^^,^^20^ As injury severity has increased over time, female patients may only visit the ED if they find their injuries severe enough to require medical attention. Since the early years of the pandemic, quarantine and other infection control practices have relaxed. While this research highlights the pandemic's impact on violence toward women, it remains unclear whether there are lasting effects in the post-pandemic era.

When evaluating for differences by racial/ethnic disparities, Black, Hispanic, Native American and Other groups were overrepresented in firearm injuries. Native American female patients had the greatest risk of visiting the ED for assault, which aligns with their greater baseline risk of lifetime violence compared to any other racial/ethnic group.^21^ Although Hispanic female patients were less likely to visit the ED for all-cause assault, this trend was reversed for firearm injuries. Hispanic female patients may be more susceptible to barriers accessing care such as possible immigration concerns.^22^^,^^23^ As such, they may wait to seek care until their injuries are more severe. Black female patients had the greatest risk of firearm injury compared to any other group. This is consistent with the Black community's disproportionate burden of gun violence and Black males having the highest rates of homicide by firearms.^24^^,^^25^ Studies indicate that Black women are particularly susceptible to intimate partner violence compared to other races and ethnicities, yet face more significant barriers to obtaining help due to factors like generally poorer health, the highest-numbers of single-mother households, the compounded effects of racial and gender discrimination, and a lack of available community resources.^26^, ^27^, ^28^, ^29^ This pattern intensified during and after the COVID-19 pandemic, with reports indicating a 49% increase in age-adjusted gun death rates among Black women between 2019 and 2023.^30^ During this period, Black women were nearly six times more likely to die by gun homicide than White women.^19^

Firearm injuries increased over the study period and were associated with stunning mortality rates. This mirrors other studies finding of global increases in firearm injuries during the pandemic, and a similar phenomenon of decreasing crime but a growth in homicide, strengthening the external validity of this analysis.^31^^,^^32^ Many of these increases were experienced during the initial years of the pandemic, with national estimates through 2021 finding an increase of 34.3% excess non-fatal firearm injuries (95% empirical confidence interval [eCI] 26.1%–41.1%) and a 28.4% increase in excess firearm deaths (95% eCI, 12.9%–46.2%).^33^ National estimates of firearm injury rates did not decrease during the pandemic until 2022, but still remained higher than pre-pandemic levels.^34^ Even during re-opening phases later in the pandemic, firearm injuries, deaths and mass-shootings increased according to data from the Gun Violence Archive.^35^ While this data set does not provide information about the assailant, another study overlapping during this study period found that lethal cases of intimate partner violence involving firearms increased by 57.6%.^36^ Poverty is also well-associated with increases in firearm injuries, which is consistent with our study, finding associations with firearm-related assaults and lower insurance status and lower quartiles of median income by ZIP code.^37^ Risk factors linked to firearm injuries such as neighborhood characteristics, psychological comorbidities, educational attainment, income, and social support overlap with those associated with violence and assault.^8^^,^^9^^,^^16^^,^^25^^,^^37^ During the pandemic, surges in gun purchasing were noted, finding half of new gun owners were female (50% in 2019 and 47% in 2020–2021).^38^ Furthermore, states with excess firearm purchases during March–July 2020 found corresponding increases in the risk of domestic violence in April 2020 (RR: 2.60; 95% CI 1.32–5.93) and May 2020 (RR: 1.79; 95% CI 1.19–2.91).^39^ Given the association, future work may target confirming these findings prospectively.

Although it appears emergency department visits due to assault are decreasing amongst female patients, this may be in part due to under self-reporting. Despite the increase in awareness of assault and violence against women in recent years, there is still a large burden amongst poorer, younger women who would benefit from more resources and support.^40^^,^^41^ Our study can help inform efforts to better address gaps in healthcare services, enhance early detection efforts, and provide comprehensive care for survivors of gender-based violence. Additionally, it can also drive policy decisions on funding for both emergency healthcare services and programs focused on gender-based violence prevention and recovery. Interventions targeting highly vulnerable subgroups of women, including those with concurrent substance use disorder while simultaneously combining IPV prevention show promise.^40^ Limited data analyzing father-focused interventions during pregnancy and the early parenting period to reduce IPV shows some benefit.^41^ A multisectoral approach to addressing violence against women that crosses disciplines is key to making meaningful change in this area.

Our results reinforce the association between firearm presence and female homicide noted previously.^28^ Critical in reducing both sex-based and race-based gun violence disparities are prohibitions on firearm possessions. For instance, federal restrictions have resulted in a 28% reduction in firearm intimate partner homicides for Black victims.^42^ State-level restrictions with relinquishment provisions have been associated with a 16% reduction in firearm partner homicide of white victims.^43^ The Supreme Court upheld the federal law prohibiting those under domestic violence restraining orders from possessing firearms in *United States v. Rahimi*.^44^ Thus, when an individual has been “found by a court to pose a credible threat to the physical safety of another,” that person may be temporarily disarmed.^44^ State laws are equally critical because they can close gaps in the federal law such as in the “boyfriend loophole,” allowing those not living with victims but with a history of intimate partner violence to also have access to firearms restricted. Our data potentially support Extreme Risk Protection Orders (ERPO) as 75% of those placed under ERPO had a history of interpersonal violence.^45^ These orders can be a vital tool in preventing firearm-related harm among vulnerable populations.^43^^,^^45^ However, enforcement remains inconsistent, and mechanisms for safe firearm relinquishment are often lacking.^43^^,^^45^

Encounter level data is provided by the care facility by means of the discharge summary. Per the HCUP documentation, to be included as an injury in the dataset (I10_INJURY), specific ICD-10 CM codes (2019 and prior) had to match. Beginning in 2020, the Clinical Classification Software Refined (CCSR) for ICD-10 CM had to align with the 2020 ICD-10-CM Injury Diagnosis Framework for Categorizing Injuries by Body Region and Nature of Injury. Physicians and other healthcare providers may present injuries, or the patient may self-report during the encounter; however injuries must be documented in the discharge summary and meet the HCUP definition of injury to be included here.^14^ Nuances with biologic sex and gender identity may not be sufficiently granular as any other sex other than non-female, non-male is coded as missing by HCUP. In general, assault is likely underreported, considering patient concerns for retaliation. This analysis relies on ED encounters. Women who do not seek medical attention in the ED are not represented here, also contributing to lower estimates. Additionally, the relationship of the patient to their assailant is not included in this data set.

The initial logistic regression was affected by sparse-data bias and quasi-complete separation, which inflated the odds ratios for several variables. We therefore employed a survey-weighted Poisson regression with a log link to estimate RRs. Although the direction and statistical significance of the key findings were consistent across models, the RRs for these specific categories remained elevated; therefore, the magnitudes of these point estimates should be interpreted with caution.

### Conclusion

This study provides a cross-sectional analysis of female assault victims presenting to the ED between 2018- and 2021. While ED visits due to assault declined, firearm-related injuries increased and were associated with significantly higher mortality rates, particularly among minoritized female patients. While younger, uninsured, and impoverished female patients shouldered a disproportionate burden, no racial, ethnic, or socioeconomic groups were spared. These results underscore the urgent need for targeted interventions, particularly around injury prevention, to reduce the incidence and severity of assault-related injuries and deaths.

## Contributors

SC: Conceptualization, data curation, formal analysis, methodology, writing (original draft), writing (review & editing), decision to submit manuscript.

IU: Methodology, writing (original draft), writing (review & editing).

MU: Writing (original draft), writing (review & editing).

OA: Conceptualization, data curation, formal analysis, methodology, validation, writing (original draft), writing (review & editing).

TX: Data curation, formal analysis, methodology, validation, visualization, writing (original draft), writing (review & editing).

WL: Methodology, conceptualization, writing (original draft), writing (review & editing).

## Data sharing statement

The source data for this analysis is available from HCUP (https://hcup-us.ahrq.gov/nedsoverview.jsp). The statistical analysis plan can be shared upon request.

## Declaration of interests

WL has received funding from the National Institutes of Health (NIH) and the American Board of Family Medicine Foundation. WL declares consulting fees for the American Academy of Family Physicians and stock/stock options with MedirAI. OA reports funding from the NIH and National Academies of Science and Engineering and Medicine (NASEM), payment or honoraria from Abt Associates, and received support from Academy Health for attending meetings and/or travel. OA has participated in a data safety monitoring board or advisory board for BRAIN Health Equity. OA holds a leadership role in the Disasters and Older Adults Interest Group of Academy Heath and reports stock/stock options with United Healthcare, Meta, Carnival Corp, and Boeing. OA is the Direct of the Humana Institute, an academic industry partnership with Human Inc. SC reports funding from the University of Houston, University of Texas Medical Branch Claude D. Pepper Older Americans Independence Center, NASEM, and the Humana Institute. SC is a member of the Harris County Medical Society and has served in leadership roles.IU and MU report no conflict of interests.
