# Supplementary material for: US emergency department visits by women due to assault (2018–2021): a retrospective cross-sectional analysis
Source: Lancet Reg Health Am. 2026 Jan 6;54:101343. doi: 10.1016/j.lana.2025.101343 (PMC12811418; doi:10.1016/j.lana.2025.101343)
Supplement: Supplementary Tables [file mmc1.docx]

**Title: US Emergency Department Visits by Women Due to Assault (2018-2021): A Retrospective Cross-Sectional Analysis**

**Supplementary Material**

Table of Contents:

Supplementary Table 1………………………………………………………………………………………………………………………………………………………………………………………Page 2

Supplementary Table 2………………………………………………………………………………………………………………………………………………………………………………………Page 4

**Supplementary Table 1:** Adjusted Logistic Regression Model Assessing the Odds of Female Adult Patients Visiting the Emergency Department Due to a) All-Cause Assault Compared to General Adult Female ED Population and b) Assault by Firearm Compared to All Other Adult Female Assault Patients

|  | Odds of Female Adult Patients Visiting the Emergency Department Due to Assault Compared to General Adult Female ED Population Visiting the Emergency Department | | Odds of Female Adult Patients Visiting the Emergency Department Due to Firearm Injury Compared to Adult Female ED Population Visiting the Emergency Department for Assault | |
| --- | --- | --- | --- | --- |
| **Variable** | **Adjusted Odds Ratio** | **95% Confidence Interval** | **Adjusted Odds Ratio** | **95% Confidence Interval** |
| *Age* | 0.97 | 0.97 – 0.97^α^ | 0.97 | 0.97 – 0.97^β^ |
| *Year* | 0.97 | 0.96 – 0.97 | 1.22 | 1.16 – 1.27 |
|  |  |  |  |  |
| *Quartile of Median ZIP Code* |  |  |  |  |
| 1^st^ | 1.11 | 1.10 – 1.13 | 1.88 | 1.62 – 2.17 |
| 2^nd^ | 0.96 | 0.94 – 0.97 | 1.35 | 1.15 – 1.59 |
| 3^rd^ | 0.96 | 0.95 – 0.98 | 1.26 | 1.07 – 1.49 |
|  |  |  |  |  |
| *Death* |  |  |  |  |
| ED | 1.04 | 0.89 – 1.21 | 379.00 | 266.15 – 539.71 |
| Hospital | 1.34 | 1.19 – 1.50 | 5.92 | 4.40 – 7.98 |
|  |  |  |  |  |
| *Payer* |  |  |  |  |
| Medicare | 1.24 | 1.21 – 1.26 | 0.36 | 0.29 – 0.45 |
| Medicaid | 2.06 | 2.03 – 2.08 | 0.78 | 0.70 – 0.86 |
| Uninsured/Self-Pay/Other | 2.76 | 2.73 – 2.80 | 0.86 | 0.77 – 0.96 |
|  |  |  |  |  |
| *Non-Metropolitan* | 0.81 | 0.80 – 0.82 | 1.02 | 0.90 – 1.15 |
|  |  |  |  |  |
| *ED Disposition* |  |  |  |  |
| Admit | 0.52 | 0.51 – 0.53 | 20.60 | 18.87 – 22.52 |
| Transfer | 1.16 | 1.12 – 1.21 | 8.94 | 7.56 – 10.58 |
| Death | -- | -- | -- | -- |
|  |  |  |  |  |
| *Race* |  |  |  |  |
| Black | 1.29 | 1.28 – 1.31 | 4.74 | 4.29 – 5.24 |
| Hispanic | 0.81 | 0.80 – 0.82 | 1.96 | 1.71 – 2.25 |
| Asian/Pacific Islander | 0.96 | 0.94 – 1.00 | 0.96 | 0.63 – 1.46 |
| Native American | 2.73 | 2.64 – 2.82 | 0.95 | 0.63 – 1.44 |
| Other | 1.09 | 1.06 – 1.11 | 1.97 | 1.56 – 2.47 |

REF = reference group. α the original 95% confidence interval is 0.9685 - 0.9696. β the original 95% confidence interval is 0.9648 - 0.9743.

**Supplementary Table 2:** Adjusted Logistic Regression Model Assessing the Odds of Female Adult Patients Visiting the Emergency Department Due to a) All-Cause Assault Compared to General Adult Female ED Population and b) Assault by Firearm Compared to All Other Adult Female Assault Patients By Age

| **Variable** | **Adjusted Odds Ratio for ED Visit Due to Assault**  **(95% CI)** | **Adjusted Odds Ratio for ED Visit Due to Firearm Injury (95% CI)** | **Adjusted Odds Ratio for ED Visit Due to Assault**  **(95% CI)** | **Adjusted Odds Ratio for ED Visit Due to Firearm Injury (95% CI)** | **Adjusted Odds Ratio for ED Visit Due to Assault**  **(95% CI)** | **Adjusted Odds Ratio for ED Visit Due to Firearm Injury (95% CI)** | **Adjusted Odds Ratio for ED Visit Due to Assault (95% CI)** | **Adjusted Odds Ratio for ED Visit Due to Firearm Injury (95% CI)** |
| --- | --- | --- | --- | --- | --- | --- | --- | --- |
|  | **18-29 Years** | | **30-39 Years** | | **40-49 Years** | | **50+ Years** | |
| *Age* | 0.99  (0.98 – 0.99) | 0.96  (0.94 – 0.97) | 0.98  (0.98 – 0.99) | 0.98  (0.96 – 1.01) | 0.97  (0.62 – 0.97) | 0.97  (0.94 – 1.01) | 0.95  (0.95 – 0.95) | 0.95  (0.94 – 0.97) |
| *Year* | 0.95  (0.95 – 0.96) | 1.24  (1.16 – 1.32) | 0.97  (0.96 – 0.98) | 1.21  (1.10 – 1.32) | 0.99  (0.98 – 1.00) | 1.22  (1.07 – 1.40) | 0.99  (0.98 – 1.00) | 1.12  (0.97 – 1.29) |
|  |  |  |  |  |  |  |  |  |
| *Quartile of Median Income ZIP Code* |  |  |  |  |  |  |  |  |
| 1^st^ | 1.01  (0.99 – 1.03) | 1.86  (1.51 – 2.30) | 1.17  (1.14 – 1.20) | 1.61  (1.21 – 2.13) | 1.15  (1.11 – 1.19) | 2.56  (1.66 – 3.94) | 1.17  (1.14 – 1.21) | 1.90  (1.22 – 2.97) |
| 2^nd^ | 0.91  (0.89 – 0.93) | 1.31  (1.04 – 1.65) | 0.99  (0.96 – 1.02) | 1.20  (0.88 – 1.63) | 0.98  (0.94 – 1.02) | 1.68  (1.06 – 2.68) | 0.96  (0.92 – 0.99) | 1.61  (1.00 – 2.59) |
| 3^rd^ | 0.94  (0.91 – 0.96) | 1.40  (1.10 – 1.77) | 0.98  (0.95 – 1.01) | 1.10  (0.80 – 1.530 | 0.95  (0.92 – 0.99) | 0.88  (0.52 – 1.50) | 0.96  (0.93 – 1.00) | 1.41  (0.85 – 2.32) |
| 4^th^ | REF | REF | REF | REF | REF | REF | REF | REF |
|  |  |  |  |  |  |  |  |  |
| *Death* |  |  |  |  |  |  |  |  |
| In ED | 3.24  (2.50 – 4.20) | 426.52  (230.66 – 788.69) | 1.92  (1.45 – 2.55) | 314.81  (171.58 – 577.58) | 0.71  (0.44 – 1.13) | 830.64  (221.04 – 3121.45) | 0.45  (0.33 – 0.63) | 256.91  (116.94 – 564.43) |
| In Hospital | 5.09  (3.81 – 6.80) | 13.53  (6.45 – 28.37) | 1.57  (1.10 – 2.24) | 9.16  (4.62 – 18.15) | 0.88  (0.59 – 1.31) | 8.09  (3.08 – 21.22) | 1.18  (1.03 – 1.36) | 3.06 (1.63 – 5.75) |
| Did not die | REF | REF | REF | REF | REF | REF | REF | REF |
|  |  |  |  |  |  |  |  |  |
| *Payer* |  |  |  |  |  |  |  |  |
| Medicare | 1.45  (1.39 – 1.53) | 0.61  (0.38 – 0.97) | 1.96  (1.89 – 2.05) | 0.25  (0.15 – 0.42) | 1.96  (1.88 – 2.04) | 0.36  (0.23 – 0.58) | 1.62  (1.57 – 1.68) | 0.36  (0.25 – 0.52) |
| Medicaid | 1.59  (1.56 – 1.61) | 0.94  (0.82 – 1.09) | 2.37  (2.32 – 2.42) | 0.77  (0.63 – 0.95) | 2.64  (2.56 – 2.72) | 0.60  (0.45 – 0.79) | 2.69  (2.61 – 2.78) | 0.40  (0.28 – 0.56) |
| Uninsured/Self-Pay/Other | 2.18  (21.4 – 2.22) | 1.00  (0.85 – 1.17) | 3.12  (3.05 – 3.20) | 0.87  (0.69 – 1.08) | 3.20  (3.11 – 3.31) | 0.67  (0.50 – 0.91) | 3.56  (3.44 – 3.68) | 0.60  (0.42 – 0.86) |
| Private | REF | REF | REF | REF | REF | REF | REF | REF |
|  |  |  |  |  |  |  |  |  |
| *Location Classification* |  |  |  |  |  |  |  |  |
| Metropolitan | REF | REF | REF | REF | REF | REF | REF | REF |
| Non-Metropolitan | 0.84  (0.83 – 0.86) | 0.94  (0.78 – 1.24) | 0.85  (0.83 – 0.87) | 0.99  (0.78 – 1.27) | 0.82  (0.79 – 0.84) | 0.94  (0.68 – 1.31) | 0.72  (0.70 – 0.75) | 1.60  (1.14 – 2.24) |
|  |  |  |  |  |  |  |  |  |
| *ED Disposition* |  |  |  |  |  |  |  |  |
| Did Not Die | Ref | Ref | Ref | Ref | Ref | Ref | Ref | Ref |
| Admit | 0.48  (0.46 – 0.50) | 27.25  (24.15 – 30.75) | 0.48  (0.46 – 0.50) | 19.33  (16.38 – 22.82) | 0.48  (0.46 – 0.50) | 14.04  (11.14 – 17.69) | 0.64  (0.62 – 0.66) | 10.47  (7.87 – 13.93) |
| Transfer | 1.63  (1.54 – 1.73) | 9.11  (7.24 – 11.45) | 1.33  (1.23 – 1.43) | 10.26  (7.50 – 14.04) | 1.09  (0.99 – 1.19) | 7.28  (4.37 – 12.12) | 0.74  (0.68 – 0.80) | 6.19  (3.41 – 11.27) |
| Die | -- | -- | -- | -- | -- | -- | -- | -- |
|  |  |  |  |  |  |  |  |  |
| *Race* |  |  |  |  |  |  |  |  |
| White | REF | REF | REF | REF | REF | REF | REF | REF |
| Black | 1.54  (1.52 – 1.56) | 5.03  (4.30 – 5.88) | 1.21  (1.18 – 1.23) | 5.34  (4.39 – 6.50) | 1.04  (1.01 – 1.06) | 4.22  (3.25 – 5.47) | 1.16  (1.13 – 1.19) | 3.76  (2.87 – 4.92) |
| Hispanic | 0.96  (0.94 – 0.97) | 1.88  (1.54 – 2.30) | 0.77  (0.75 – 0.79) | 1.88  (1.42 – 2.50) | 0.66  (0.64 – 0.68) | 2.58  (1.84 – 3.61) | 0.68  (0.66 – 0.71) | 2.09  (1.38 – 3.15) |
| Asian/Pacific Islander | 0.92  (0.87 – 0.97) | 1.01  (0.52 – 1.94) | 0.90  (0.84 – 0.95) | 0.81  (0.35 – 1.85) | 0.90  (0.83 – 0.97) | 0.68  (0.21 – 2.24) | 1.20  (1.13 – 1.27) | 1.30  (0.54 – 3.12) |
| Native American | 2.87  (2.71 – 3.03) | 1.24  (0.68 – 2.25) | 2.71  (2.55 – 2.87) | 1.14  (0.56 – 2.29) | 2.50  (2.32 – 2.70) | 0.56  (0.17 – 1.83) | 2.30  (2.11 – 2.52) | -- |
| Other | 1.21  (1.17 – 1.25) | 2.14  (1.55 – 2.96) | 0.98  (0.94 – 1.03) | 2.68  (1.78 – 4.03) | 0.97  (0.91 – 1.02) | 1.48  (0.62 – 3.50) | 1.17  (1.10 – 1.24) | 0.66  (0.26 – 1.67) |
